# Coordinating resources for prospective medication risk management of older home care clients in primary care: procedure development and RCT study design for demonstrating its effectiveness

**DOI:** 10.1186/s12877-018-0737-z

**Published:** 2018-03-16

**Authors:** Terhi Toivo, Maarit Dimitrow, Juha Puustinen, Eeva Savela, Katariina Pelkonen, Valtteri Kiuru, Tuula Suominen, Sirkka Kinnunen, Mira Uunimäki, Sirkka-Liisa Kivelä, Saija Leikola, Marja Airaksinen

**Affiliations:** 1 Faculty of Pharmacy, Division of Pharmacology and Pharmacotherapy, Clinical Pharmacy Group, University of Helsinki, Viikinkaari 5 E, P.O. BOX 56, 00014 Helsinki, Finland; 2 Satakunta Hospital District, Satakunta Central Hospital, Unit of Neurology, Sairaalantie 3, 28500 Pori, Finland; 3 1st Pharmacy of Lohja, Laurinkatu 37-41 A, 08100 Lohja, Finland; 4 City of Lohja, Services for Aged Residents, PL 71, 08101 Lohja, Finland; 5 Institute of Clinical Medicine, Department of Family Medicine, University of Turku, 20014 Turku, Finland

**Keywords:** Medication risk management, Home care, Older adults, Coordinated procedure, Randomized controlled trial

## Abstract

**Background:**

The magnitude of safety risks related to medications of the older adults has been evidenced by numerous studies, but less is known of how to manage and prevent these risks in different health care settings. The aim of this study was to coordinate resources for prospective medication risk management of home care clients ≥ 65 years in primary care and to develop a study design for demonstrating effectiveness of the procedure.

**Methods:**

Health care units involved in the study are from primary care in Lohja, Southern Finland: home care (191 consented clients), the public healthcare center, and a private community pharmacy. System based risk management theory and action research method was applied to construct the collaborative procedure utilizing each profession’s existing resources in medication risk management of older home care clients. An inventory of clinical measures in usual clinical practice and systematic review of rigorous study designs was utilized in effectiveness study design.

**Discussion:**

The new coordinated medication management model (CoMM) has the following 5 stages: 1) practical nurses are trained to identify clinically significant drug-related problems (DRPs) during home visits and report those to the clinical pharmacist. Clinical pharmacist prepares the cases for 2) an interprofessional triage meeting (50–70 cases/meeting of 2 h) where decisions are made on further action, e.g., more detailed medication reviews, 3) community pharmacists conduct necessary medication reviews and each patients’ physician makes final decisions on medication changes needed. The final stages concern 4) implementation and 5) follow-up of medication changes. Randomized controlled trial (RCT) was developed to demonstrate the effectiveness of the procedure.

The developed procedure is feasible for screening and reviewing medications of a high number of older home care clients to identify clients with severe DRPs and provide interventions to solve them utilizing existing primary care resources.

**Trial registration:**

The study is registered in the Clinical Trials.gov (NCT02545257). Registration date September 9 2015.

## Background

The magnitude of safety risks related to medications of the older adults has been evidenced by numerous studies [1, 2], but less is known of how to manage and prevent these risks in different health care settings. Since the 1990s, both explicit and implicit criteria have been established to decrease prescribing of potentially inappropriate medicines (PIMs) for the older adults [3–5]. These criteria are useless unless they are implemented routinely throughout health care. Recently, the implementation has been facilitated through electronic databases, software applications and clinical decision support systems (CDSS), which have dramatically evolved over the last decade [6, 7]. The databases and CDSS systems can prospectively detect PIMs and other medication safety risks, but qualified healthcare professionals are needed to make the final decision using clinical judgement based on comprehensive patient information. Efficient use of these modern tools and skill-sets requires coordinated medication management processes in different healthcare settings.

**Fig. 1:**
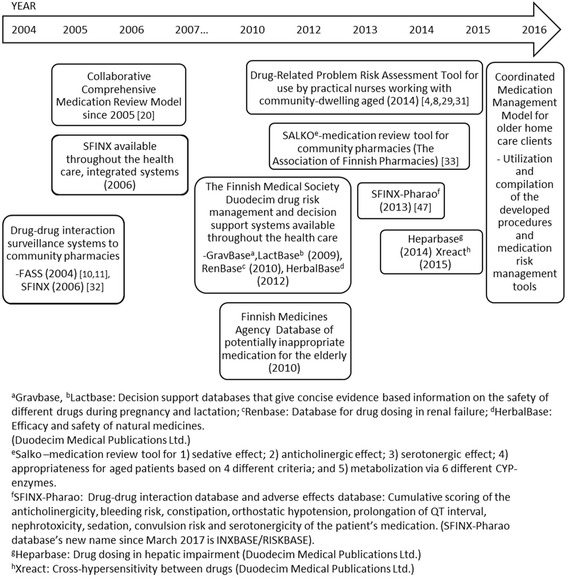
Medication risk management tools and databases launched in Finland since 2004 and currently widely available in health care and community pharmacies

Finland is one of the countries with advanced national health portals, databases and prospective screening systems for managing drug-related risks (Fig. 1) [8–11]. Within less than 20 years, a wide range of medication risk management tools have been developed, with the Finnish Medical Society Duodecim playing a major role in their development [9]. These tools are widely available in Finnish health care, including community pharmacies.

Even though these innovative prospective medication risk management tools and databases are widely available in Finland, they do not form an integrated medication management process. Health care providers and units work independently, without coordination, and no one seems to have clear responsibility for identifying and solving individual patients’ drug-related problems (DRPs) [12].

The aim of this study was to integrate risk assessment tools, procedures and databases available in Finland to form a coordinated medication management model (CoMM) involving home care nurses and practical nurses (PNs), physicians and community pharmacists in the medication process of older home care clients. A study design was also developed for assessing the effectiveness of CoMM.

## Methods/Design

### Context of the study and its importance for ongoing social and health services reform in Finland

Health care services in Finland are primarily funded and organized by municipalities [13]. The services are divided into primary care and specialized care services. The municipalities and individual residents may acquire services from the public and private health care providers or the third sector. Medicine supply and related pharmaceutical services for outpatients are provided by private community pharmacies. Currently, the Finnish health care system is undergoing a massive reform [14]. The goal is to improve integration between primary and specialized care and integrate social and health services from the clients’ perspective. Integration concerns service provision and funding as they both are currently fragmented.

Long-term home care services for the older adults are a critically important part of health care delivery in Finland as in many other countries [15, 16]. Currently they are mostly based on regular, even daily, encounters with home care PNs, coordinated by home care nurses. The allocation of physicians’ time for clients is limited and will become even more limited in the future as the proportion of the older adults is increasing [17]. This is putting more pressure on developing new collaborative procedures for monitoring benefits and risks of medication therapies. As part of reorganizing the care PNsʼ involvement in monitoring medication risks and benefits could be enhanced, but PNs have the very basics of relevant pharmacotherapy [18]. A need for enhancing pharmacists’ involvement in medication management of the older adults is identified [12, 19].

The aim of this study was to coordinate resources for prospective medication risk management of home care clients ≥ 65 years in primary care and to develop a study design for demonstrating effectiveness of the procedure.

### Study setting: participating organizations, health professionals and home care clients

This study is conducted within publicly funded primary care in Lohja, a municipality in Southern Finland with 47,000 inhabitants. Health care units involved in the study are: Lohja Home Care Unit, Lohja Health Center, and a private community pharmacy (Lohja 1st Pharmacy). A clinically trained researcher (TT) from the research group coordinated the development of CoMM and the design for the effectiveness study in close cooperation with the health care providers involved in home care in Lohja.

Lohja Home Care Unit is divided into five service areas, each having a leading nurse, nurses and PNs who mostly conduct home visits. Nurses consult physicians (working in the Lohja Health Center) as needed, but the physicians meet the patients infrequently. Community pharmacists from the Lohja 1st Pharmacy had primarily a standard BSc(Pharm) or MSc(Pharm) degree with long-term in-house training for geriatric pharmacotherapy and managing DRPs in the older adults, but a pharmacist with accreditation in comprehensive medication reviews (CMRs) [20] was available as needed. All these pharmacists are termed “pharmacists” in this report.

#### Participants

Home care clients were recruited in the study by nurses and PNs. They individually approached their clients and/or their closest proxy and invited them to participate in the study. Announcements in the local newspaper were also used. The recruitment process was carried out between September 2015 and December 2015. The inclusion criteria were: 1) ≥ 65 years old, home-dwelling resident; 2) receives regular home care from the city of Lohja; 3) uses at least one medicine; 4) voluntary participation, written informed consent to participate in the study given by participant or closest proxy.

### Study design

This study applied action research method to develop the CoMM. Action research method is increasingly being used in health services research [21, 22]. When applying this method, the researcher works explicitly with and for people rather than undertaking research on them [22]. The model development was theoretically guided by Reason’s systems-based risk management theory on preventing human errors [23], complemented by Hepler and Strand’s basic principles of identifying, solving and preventing DRPs [24]. Clyne’s model was applied for categorization of comprehensiveness of medication reviews [25].

#### Development process of CoMM (intervention) and study design for demonstrating its effectiveness

The goal was to construct a collaborative procedure which utilizes each profession’s resources in a rational way. The coordination of the use of the risk management tools and resources illustrated in Fig. 1 was part of the process.

**Fig. 2:**
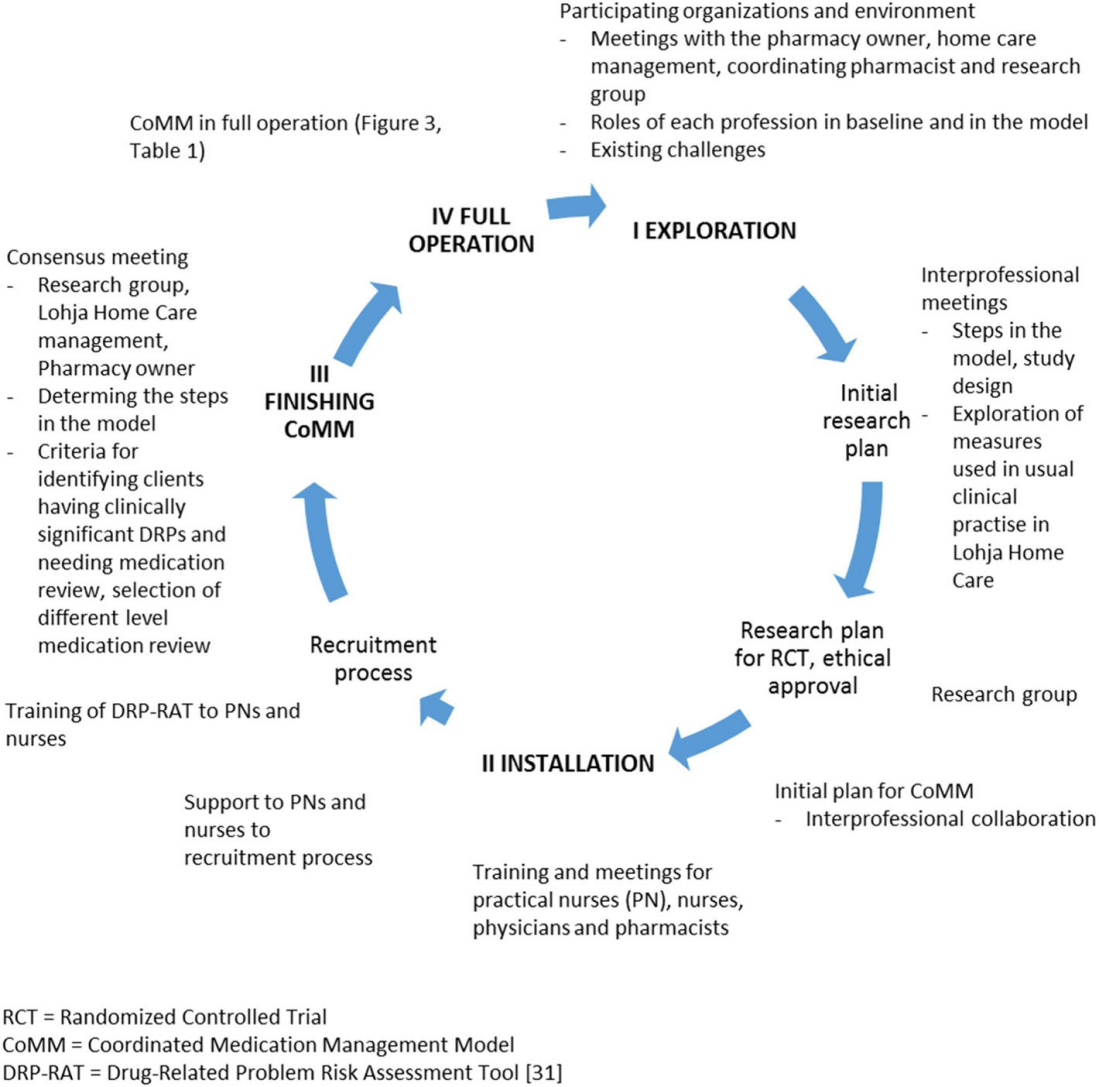
Development process of coordinated medication management model (CoMM) using action research method (modified from Lewin 1946 [21], Meyer 2000 [22])

The development process encompassed four main steps (Fig. 2). During each step, the coordinating pharmacist/researcher (TT) worked closely with the home care nurses and PNs, their manager (nurse), physicians involved in home care and the community pharmacists.

Exploration step (I) included orientation to each organization’s current medication management practices targeted to older home care clients. It also covered identification of medication management tools and procedures applied locally in Lohja compared to those generally available in Finland (Fig. 1). Actual tasks and responsibilities for each professional were defined in regular joint meetings with the coordinating pharmacist (TT), the pharmacy owner (ES) and the responsible nurse of home care service area (KP).

An inventory of clinical measures, used in routine clinical practice in Lohja Home Care, was conducted to include them as outcome measures in the effectiveness study design protocol. The ECHO model, which covers economic, clinical and humanistic outcomes, was applied for their selection and categorization [26]. Systematic review of Kiiski et al. (2016) [27], and other previous literature were applied to learn about experiences of rigorous study designs for assessing effectiveness of collaborative medication management models for the older home care clients.

Installation step (II) was to prepare the participating organizations for the CoMM. Home care nurses, PNs, physicians and pharmacists were informed prior to the study and encouraged continuously to comment on the model construction. Personnel training sessions needed to support the model construction were jointly planned with the researchers and home care management. The coordinating pharmacist organized trainings for PNs related to the recruitment process, medication reconciliation [28] and use of clinical tests. PNs were also trained on the content and use of the Drug-Related Problem Risk Assessment Tool (DRP-RAT) [8, 29] and about Lohja Home Care Unit’s principles in medication management [30].

Finishing the CoMM (III) aimed to decide the way to solve the identified clinically significant DRPs and allocate medication reviews according to the severity of the DRPs. In the consensus meeting home care physicians, home care managers, leading nurses, community pharmacists, coordinating pharmacist and the research group discussed to agree on tasks and to set up criteria for medication reviews and their comprehensiveness. Steps and procedures related to patient information transfer, interprofessional collaboration and adequate follow-ups were discussed.

After finishing the development of CoMM, its full operation started (IV). The coordinating pharmacist worked closely with the home care practitioners and community pharmacists, facilitating integration between stages and tasks of the health care providers involved. This enabled reflection of the model’s feasibility.

Study design for assessing effectiveness of CoMM was developed and selection of outcome measures was made in collaboration with Lohja Home Care and the research group utilizing published evidence on assessing effectiveness of medication review procedures. Selection was made considering study purposes, reliability, validity, sensitivity and specificity of measures and feasibility of carrying out the measurements in clinical practice. Measures already in use were prioritized to minimize additional work for nurses and PNs and inconvenience for the patients.

### Description of developed CoMM for home care clients (intervention)

**Fig. 3:**
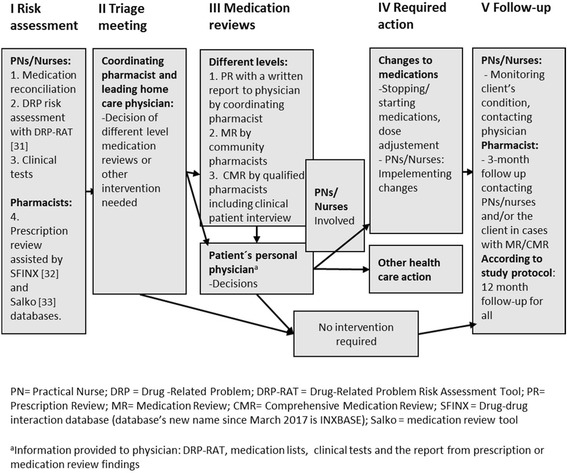
Developed coordinated medication management model (CoMM) for older home care clients

The developed CoMM consists of five main stages in which clinically significant DRPs can be identified and solved using collaborative procedures and medication reviews (Fig. 3, Table 1). PNs were trained to observe potential medication risks on routine home visits more systematically than before and to report detected clinically significant DRPs to the coordinating pharmacist (Fig. 3: Stage I: Risk Assessment). The coordinating pharmacist prepared the cases for the triage meeting (Fig. 3: Stages I and II), in which the leading home care physician and the coordinating pharmacist decided on further actions for clients with clinically significant DRPs (50–70 cases per triage meeting of two hours). The actions included more comprehensive medication reviews according to the needs of the clients involving their own physicians and nurses/PNs. In most complicated cases also home visit and the client’s clinical interview were conducted (Fig. 3: Stage III). Based on the information gathered in Stages I-III client’s personal physician made decisions on required actions (Fig. 3: Stage IV) and follow-up was organized (Fig. 3: Stage V).

**Table 1:** Agreed tasks of each healthcare professional and tools used in the coordinated medication management model (CoMM)

Healthcare professionals	Tasks in the coordinated medication management model	Tools used
Home care nurses (practical nurses, nurses)	Medication reconciliation Medication risk assessments Clinical tests to assess clients’ functioning and disability (at baseline, 12 and 24 month follow-up)	Medication lists, usual home visits Clinical interviews with the DRP-RAT a) Measures used in usual clinical practice: functional ability (RAVA) [37], physical performance (The five-times-sit-to-stand test) [38, 39], cognitive functioning (MMSE) [40], depression (GDS-15) [41] and malnutrition (MNA) [42]. b) Added measures: difficulties related to urination (UDI-6) [43], orthostatic hypotension (3 min test) [44] and alcohol use (AUDIT-C) [45].
	Implementing medication changes and monitoring their outcomes	Regular home visits as usual Informing physicians when needed
Community pharmacists	Prescription review (PR)	Clinically significant drug-drug interactions (DDIs) (SFINX) [32] Potentially inappropriate medicines (PIMs) according to Beers 2015 criteria [46], anticholinergic and serotonergic loads of medicines (Salko) [33].
	Medication review (MR)	Patient information: medication list, DRP-RAT and glomerulus filtration rate (GFR) results Other tools used: SFINX (DDIs), Pharao (Cumulative scoring of the anticholinergicity, bleeding risk, constipation, orthostatic hypotension, prolongation of QT interval, nephrotoxicity, sedation, convulsion risk and serotonergicity of the patient’s medication) [47], Salko (PIMs) [33], Renbase (Renal function and appropriateness of doses/medicines used) [9].
	Comprehensive medication review (CMR) conducted by a qualified pharmacist (TT, SL)	Patient information: medication list, DRP-RAT and GFR results, diagnosis, laboratory test results. Tools used: As in MR, complemented by client’s clinical interview [20, 48]
Coordinating pharmacist	Trainings of the PNs for the recruitment process, CoMM and use of DRP-RAT (MD)	Meetings, discussions, personal guidance, DRP-RAT training [29]
	Coordinating and organizing processes for CoMM	Constructing the CoMM structure through observations, meetings, contacts and negotiations with organizations, health care professionals, researchers and home care clients involved, organizing processes and interactive training, providing training, guidance and feedback, reflecting the literature and guidelines on geriatric care and pharmacotherapy
	Preparing triage meetings with the leading home care physician to decide on actions for clients with clinically significant DRPs	Prescription review findings (from SFINX and Salko databases) and DRP-RAT results.
Leading home care physician	Triage meetings with the coordinating pharmacist to decide on actions for clients with clinically significant DRPs (50–70 cases per triage meeting of 2 h)	Prescription review findings (from SFINX and Salko databases) and DRP-RAT results.
Client’s personal physician	Case-conferences with pharmacists concerning clients with clinically significant DRPs identified in MR and CMR. Decisions on the medication changes and how they will be implemented.	Medication lists accomplished with the SFINX and Salko data, DRP-RAT results, results from the clinical tests, laboratory test (GFR), MR and CMR report, including client’s clinical interview.

*Abbreviations*: *DRP-RAT* Drug-Related Problem Risk Assessment Tool [31], *MMSE* Mini Mental State Examination [40], *GDS-15* Geriatric Depression Scale [41], *MNA* The Mini Nutritional Assessment [42], *UDI-6* Urinary Distress Inventory [43], *AUDIT-C* Alcohol Use Disorder Identification Test, version C [45], *SFINX* Drug-drug interaction database’s new name since March 2017 is INXBASE, *Pharao* adverse effects database’s new name since March 2017 is RISKBASE

Collaborative tasks of each healthcare professional in the developed model (Fig. 3) are described in Table 1. Nurses and PNs had a key role in clinical follow-up and identifying clients with clinically significant DRPs through gathering and bringing information about clients’ symptoms and signs by DRP-RAT. Community pharmacists’ pharmacotherapeutic skills were utilized in medication reviews at Stage III (Fig. 3). Physicians’ resources were allocated for clinical decision making at the triage stage (Stage II) and for deciding on actions for clients with complicated DRPs analyzed more in detail in prescription review (PR), medication review (MR) or comprehensive medication review (CMR). (Stage IV). The coordinating pharmacist had a key role in organizing and coordinating medication management processes between the fragmented organizations involving different health care providers, and in preparing and participating in the triage meetings followed by different level medication reviews.

### Description of study design for assessment of CoMM’s outcomes and effectiveness

Randomized controlled trial (RCT) was selected for assessing the effectiveness of CoMM.

#### Trial design

The CoMM study is a randomized controlled superiority trial with two parallel groups, a 1:1 randomization (intervention and control).

#### Randomization

Participants were randomized to an intervention group (IG) and a control group (CG) receiving standard home care. To prevent contamination between IG and CG and subsequent dilution of the intervention, participants were randomized by home care areas (2 areas to the IG, 3 areas to the CG) since each home care area has its own nursing staff. The randomization was performed by sealed envelopes. The study is considered as open-label. The IG (*n* = 104) received the intervention (CoMM) during the first year, while the CG (*n* = 87) received standard care and received the same intervention after the first year (delayed intervention as control).

#### Participant timeline

Selected study period was 2 years with measurements at baseline (in 2015), and at 12 (in 2016) and 24 months (in the end of year 2017).

#### Sample size

The study sample was all home care clients, meeting inclusion criteria and giving voluntary informed consent, in the Lohja Home Care Unit due to the practical, administrative and financial issues. Sample size calculations were not performed.

#### Outcome measures

Selected primary outcome measures assess appropriateness of the medications used, general health status and functional ability of the older adults, but also target to specific symptoms that can be potentially caused as adverse effects of medications (Table 1). Majority of selected clinical measures were used in usual clinical practice in Lohja Home Care. Medication-specific measures include assessment of clinically significant DRPs by using DRP-RAT [31] and electronic screening tools (SFINX, Salko) for identifying PIMs for the older adults, anticholinergic and serotonergic load and clinically significant drug-drug interactions (DDIs) [32, 33]. Medicines will be classified according to the anatomical therapeutic chemical (ATC) classification system [34]. Use of health services will be measured as visits to physicians, frequency of visits of home care personnel and frequency of hospital days and will be used as a secondary outcome.

#### Data collection methods and data management

Home care nurses and practical nurses conduct clinical tests and DRP-RAT –assessments, compile medication lists and deliver the data to the research coordinator (TT). Pharmacists use case report form in medication reviews and research coordinator receives the data. Data are entered into a computerized database by the research coordinator. All patients will be given a unique study number to preserve confidentiality. The collected data will be verified for accuracy, missing data, and data consistency with the documents source (medication lists, clinical test forms).

#### Statistical methods

Data will be analyzed on an intention to treat principle, including all randomized participants in the group to which they were randomly assigned. Also per protocol analysis to compare participants from intervention group with clinically significant medication changes conducted due to CMR, MR or PR, with control group will be performed.

The effectiveness of intervention compared to controls receiving standard home care will be first analyzed with unadjusted analysis. If there exists any group differences in participant characteristics or clinical outcomes at baseline, these variables will be included in adjusted analysis.

Descriptive statistics (mean, median or percentages as appropriate) will be used to summarize the participant characteristics and clinical outcomes. The comparison in the participant characteristics and clinical outcomes at baseline between groups will be done by two-sample t-test for normally distributed variables and by Mann-Whitney U-test for non-normally distributed variables. Chi-square test will be used for categorical variables.

Continuous outcomes will be analyzed with analysis of variance or covariance and repeated measures analysis of variance or covariance. Dichotomous outcomes will be analyzed by binary logistic regression and ordinal outcomes by cumulative logistic regression using generalized estimating equations to account for the correlation between the repeated measurements. Participants with baseline measurement and at least one follow-up measurement will be included in longitudinal analysis. Two-sided statistical tests with a 5% level of significance will be used.

#### Data monitoring

The research coordinator ensures the successful completion of the study, and the collection of data. She also ensures the compliance with the study protocol, the organization of the follow-up of the study participants and receives information about drop-outs.

#### Harms – End of protocol

Any harm to the participants will not be expected due to this study. Participating physicians from their own health system decide potential changes to patients’ medication according to their normal clinical practice. Participants can withdraw their consent any time.

#### Protocol amendments

Important protocol amendment would require new ethical approval. Any important amendments have not been made during the study.

#### Confidentiality

The personal information of the participants is kept in a separate file. The research coordinator ensures the protection of the confidentiality of the data. Personal information is not entered to the study database, study numbers are used instead.

#### Dissemination policy

The results of the effectiveness study will be published in a peer-reviewed journal. Separate manuscripts may be published on primary and secondary outcomes.

## Discussion

This study produced a 5-stage medication management procedure suitable for screening medications of a high number of home care clients and identifying clients with potential clinically significant DRPs. The model coordinates existing resources to prospective medication risk assessment providing also tools to solve identified DRPs. Nurses and PNs’ role in conducting DRP risk assessments, medication reconciliation and clinical tests during their usual home visits was clarified and reinforced. They also had a key role in implementing and following up medication changes. Triage meetings was a new and feasible way for allocating medication reviews according to clinical needs, but using a minimum of physician’s time. The coordinating pharmacist prepared triage meetings by summing up each client’s DRP risk information from different sources and making preliminary proposals for required actions for physician’s consideration. Community pharmacists’ contribution changed towards more clinical in the model. They conducted medication reviews and worked closer than before with nurses, PNs and physicians. In future, the coordinating role could be delegated to community pharmacists.

The model contains an adequate follow-up stage to confirm that the agreed medication changes will be actually implemented and the client’s health status monitored. This stage is often missing or omitted, but it is crucial for obtaining any benefits from DRP risk assessments and medication reviews [27].

The model focuses on clinically significant DRPs which may occur due to patient-related factors (e.g., age-related physiological alterations, co-morbidities, poor adherence), pharmacological effects of the medications (particularly adverse drug reactions (ADRs), high-risk medications) or the medication process of the client (e.g., poor medication management, infrequent follow-ups, various health care providers) [31]. These are the aspects that PNs were trained to observe during home visits by using the DRP-RAT tool as a guide in communication with their client or the proxy. Home visits were primarily conducted by clients’ own PNs who knew them. A clinically trained pharmacist conducted home visits only in cases in which risk assessment conducted by a PN indicated serious DRPs needing more detailed investigation. These cases were a minority in our data.

The CoMM development process revealed educational needs both in geriatric pharmacotherapy and understanding system-based medication risk management. These needs were identified in all participating health care professionals and community pharmacists. This kind of model development processes should include interprofessional training that support competence and practice development [35]. In our process, home care nursing staff and physicians had training on identifying clinically significant DRPs by using DRP-RAT and deprescribing [8, 31, 36]. Community pharmacists were identified to need training for conducting DRP risk assessments and taking more responsibility of the triage stage in the future. The coordinating pharmacist was a valuable resource in identifying educational needs and educating staff.

Our experience is that health care teams in home care benefit from having a coordinating pharmacist with qualifications in CMRs, geriatric pharmacotherapy and system-based medication risk management. Our study revealed that organizations and health care units involved in home care clients’ medication therapy are working independently in silos, nobody takes holistic responsibility for medications. The coordinating pharmacist was needed to facilitate construction of new processes and introduce new tools and approaches in medication management. She scheduled the progression of risk management stages (see Fig. 3) and regularly highlighted the primary goal of the project to those involved: the purpose being to find a feasible way to manage and prevent clinically significant DRPs of the home care clients, not to conduct scientific research. Practitioners involved were not used to working in such close collaboration, which was crucial for the model. Scarce availability of physicians’ resources and partly reluctant attitudes towards the new collaborative way of working complicated the arrangement of case-conferences of MRs and CMRs.

System based risk management perspectives through Reason’s Swiss Cheese Model [23] and Hepler and Strand’s model [24], to identify and prevent DRPs, were useful in guiding model development and constructing a shared understanding of medication safety and prospective medication risk management. Our study indicated that practitioners in Finnish health care are not well acquainted with systems thinking and this needs reinforcement in the future.

The strength in using an action research method [21] in model development lies in its ability to consider practical challenges and produce solutions, considering existing resources. The method contributed to the step by step construction of the CoMM model and description of the responsibilities of each professional involved in the model, which is missing in many other studies [27]. Transferring the model to other home care localities is possible, but will require long term effort from a qualified coordinator, committed personnel and managers to reach the mature stage of the collaboration that is necessary for sustainable changes in working patterns.

This study produced a RCT with a combination of outcome measures to assess general health status and functional ability of the older adults, but also targeted to symptoms suggestive of adverse effects of medications. DRP-RAT is used as an outcome measure to evaluate potential decreases in clinically significant DRPs. Medication lists are used to investigate changes in the quality of medications (e.g., use of PIMs). According to previous evidence, a selected study period of 2 years should be long enough to demonstrate potential changes in study participants’ health outcomes, use of health services and sustainability of changes made in their medications.

The developed CoMM procedure is feasible for screening and reviewing medications of a high number of older home care clients to identify clients with severe DRPs and provide interventions to solve them utilizing existing primary care resources. The coordinating pharmacist was needed to facilitate the construction of new processes and introduce new tools and approaches in medication management of the older home care clients.

## Abbreviations

ADR(s): Adverse drug reaction(s)
ATC: Anatomical therapeutic chemical
CDSS: Clinical decision support systems
CG: Control group
CMR(s): Comprehensive medication review(s)
CoMM: Coordinated medication management model
DDI(s): Drug-drug interaction(s)
DRP(s): Drug-related problem(s)
DRP-RAT: Drug-Related Problem Risk Assessment Tool
ECHO: Economic, clinical and humanistic outcomes
IG: Intervention group
MR: Medication review
PIM(s): Potentially inappropriate medicine(s)
PN(s): Practical nurse(s)
PR: Prescription review
RCT: Randomized controlled trial

## References

[1] Panagioti M, Stokes J, Esmail A (2015). Multimorbidity and patient safety incidents in primary care: a systematic review and meta-analysis. PLoS One.

[2] Tommelein E, Mehuys E, Petrovic M (2015). Potentially inappropriate prescribing in community-dwelling older people across Europe: a systematic literature review. Eur J Clin Pharmacol.

[3] Spinewine A, Schmader KE, Barber N (2007). Appropriate prescribing in elderly people: how well can it be measured and optimized? Review. Lancet.

[4] Dimitrow M, Airaksinen M, Kivelä S-L (2011). Comparison of prescribing criteria to evaluate the appropriateness of drug treatment in individuals aged 65 and older: a systematic review. J Am Geriatr Soc.

[5] Lucchetti G, Lucchetti A (2017). Inappropriate prescribing in older persons: a systematic review of medications available in different criteria. Arch Gerontol Geriat.

[6] Simon SR, Keohane CA, Amato M (2013). Lessons learned from implementation of computerized provider order entry in 5 community hospitals: a qualitative study. BMC Med Inform Decis Mak.

[7] Schiff GD, Hickman TT, Volk LA (2016). Computerised prescribing for safer medication ordering: still a work in progress. BMJ Qual Saf.

[8] Dimitrow M. Development and validation of a drug-related problem risk assessment tool for use by practical nurses working with community-dwelling aged. Doctoral Thesis, University of Helsinki, 2016. Available at: http://urn.fi/URN:ISBN:978-951-51-2618-4. Accessed 24 Sept 2017.

[9] The Finnish Medical Society Duodecim. http://www.duodecim.fi/english. Accessed 24 Mar 2017.

[10] Heikkilä T, Lekander T, Raunio H (2006). Use of an online surveillance system for screening drug interactions in prescription in community pharmacies. Eur J Clin Pharmacol.

[11] Toivo T, Mikkola J, Laine K, Airaksinen M (2016). Identifying high risk medications causing potential drug–drug interactions in outpatients: a prescription database study based on an online surveillance system. Res Soc Adm Pharm.

[12] Kallio S, Kumpusalo-Vauhkonen A, Järvensivu T (2016). Towards interprofessional networking in medication management of the aged: current challenges and potential solutions in Finland. Scand J Prim Health Care.

[13] Ministry of Social Affairs and Health. Social and Health Services (online). Available at: http://stm.fi/en/social-and-health-services. Accessed 15 Sept 2017.

[14] Ministry of Social Affairs and Health. Health, social services and regional government reform in Finland (online). Available at: http://alueuudistus.fi/frontpage. Accessed 15 Sept 2017.

[15] Teperi J, Porter ME, Vuorenkoski L et al. The Finnish health care system: a value-based perspective. Sitra Reports 82, 2009. Available at: http://www.hbs.edu/faculty/Publication%20Files/Finnish_Health_Care_System_SITRA2009_78584c8b-10c4-4206-9f9a-441bf8be1a2c.pdf. Accessed 5 May 2017.

[16] World Health Organization 2012. Home care across Europe 2012, current structure and future challenges. Available at: http://www.euro.who.int/__data/assets/pdf_file/0008/181799/e96757.pdf. Accessed 5 May 2017.

[17] Official Statistics of Finland (OSF): Population projection [e-publication]. ISSN=1798–5153. Helsinki: Statistics Finland. Available at: http://www.stat.fi/til/vaenn/index_en.html. Accessed 6 Mar 2017.

[18] Finnish National Board Of Education. Vocational qualification in social and health care, practical nurse 2010, Regulation 17/011/2010. Publications 2011:21. Available at: http://www.oph.fi/download/140436_vocational_qualification_in_social_and_healthcare_2010.pdf. Accessed 24 Sept 2017.

[19] Kumpusalo-Vauhkonen A, järvensivu T, Mäntyla A (eds.). A multidisciplinary approach to promoting sensible pharmacotherapy among aged persons -National assessment and recommendations. Finnish Medicines Agency Fimea 2016:8. (abstract in English). Available at: http://www.fimea.fi/documents/160140/1153780/KAI+8_2016.pdf/7acaeff3-999e-4749-8a47-36fbcb4db8b7. Accessed 24 Sept 2017.

[20] Leikola S. Development and Application of Comprehensive Medication Review Procedure to Community-Dwelling Elderly. Doctoral Thesis, University of Helsinki, 2012. Available at: http://urn.fi/URN:ISBN:978-952-10-7698-5. Accessed 24 Sept 2017.

[21] Lewin K (1946). Action research and minority problems. J Soc Issues.

[22] Meyer J (2000). Using qualitative methods in health related action research. Br Med J.

[23] Reason J (2000). Human error: models and management. BMJ.

[24] Hepler CD, Strand LM (1990). Opportunities and responsibilities in pharmaceutical care. Am J Hosp Pharm.

[25] Clyne W, Blenkinsopp A, Seal R: A Guide to Medication Review 2008. The National Prescribing Centre, the Medicines Partnership Programme 2.1. Available at: http://www.cff.org.br/userfiles/52%20-%20CLYNE%20W%20A%20guide%20to%20medication%20review%202008.pdf. Accessed 24 Sept 2017.

[26] Kozma CM, Reeder CE, Schulz RM (1993). Economic, clinical, and humanistic outcomes: a planning model for pharmacoeconomic research. Clin Ther.

[27] Kiiski A, Kallio S, Pohjanoksa-Mäntylä M et al. Collaborative medication management models in the rationalization of the medication therapies of the aged. Systematic review. 2016 [Finnish publication, Ministry of Social Affairs and Health, Publication 2016:12]. E-publication: http://urn.fi/URN:ISBN:978-952-00-3704-8

[28] The Joint Commission. Sentinel Event Alert, Issue 35: Using medication reconciliation to prevent errors. January 25, 2006. Available at: http://www.jointcommission.org/assets/1/18/SEA_35.pdf. Accessed 22 Mar 2017. 16463453

[29] Dimitrow MS, Leikola SN, Kivelä SL (2015). Feasibility of a practical nurse administered risk assessment tool for drug-related problems in home care. Scand J Public Health.

[30] Ministry of Social Affairs and Health. Safe Pharmacotherapy, National guide for pharmacotherapy in social and health care, an abbreviated version. Publications of the Ministry of Social Affairs and health 2009:10. Available at: http://urn.fi/URN:NBN:fi-fe201504226976. Accessed 16 June 2016.

[31] Dimitrow MS, Mykkänen SI, Leikola SNS (2014). Content validation of a tool for assessing risks for drug-related problems to be used by practical nurses caring for home-dwelling clients aged ≥65 years: a Delphi survey. Eur J Clin Pharmacol.

[32] Böttiger Y, Laine K, Andersson ML (2009). SFINX –a drug-drug interaction database designed for clinical decision support systems. Eur J Clin Pharmacol.

[33] Leikola S, Salimaki J, Teinila T et al. Salko –a medication review tool for community pharmacies. Poster, FIP Congress in Dublin, 2013. Available at: http://www.fip.org/?page=abstracts&action=generatePdf&item=8600. Accessed 24 Sept 2017.

[34] World Health Organisation (WHO), Collaborating Centre for Drug Statistics Methodology: The Anatomical Therapeutic Chemical (ATC) classification system. Available at: www.whocc.no. Accessed 18 Oct 2016.

[35] Holmström AR, Airaksinen M, Laaksonen R (2015). Introducing basic principles of medication safety: development of a three-day continuing education course for healthcare professionals. Curr Pharm Teach Learn.

[36] Page A, Clifford R, Potter K (2016). The feasibility and effect of deprescribing in older adults on mortality and health: a systematic review and meta-analysis. Br J Clin Pharmacol.

[37] RAVA -Functional Ability Test for classifying the abilities of the elderly and planning necessary services. Finnish Consulting Group. Available at: http://www.fcg.fi/eng/expertise/welfare_and_ict_services/classification_products. Accessed 16 June 2016.

[38] Csuka M, McCarty DJ (1985). Simple method for measurement of lower extremity muscle strength. Am J Med.

[39] Guralnik JM, Simonsick EM, Ferrucci L (1994). A short physical performance battery assessing lower extremity function: association with self-reported disability and prediction of mortality and nursing home admission. J Gerontol.

[40] Folstein MF, Folstein SE, McHugh PR (1975). “Minimental state”. A practical method for grading the cognitive state of patients for the clinician. J Psychiatr Res.

[41] Kurlowicz L, Greenberg SA (2007). The geriatric depression scale. Am J Nurs.

[42] Vellas B, Guigoz Y, Garry PJ (1999). The mini nutritional assessment (MNA) and its use in grading the nutritional state of elderly patients. Nutrition.

[43] Uebersax JS, Wyman JF, Shumaker SA (1995). Short forms to assess life quality and symptom distress for urinary incontinence in women: the incontinence impact questionnaire and the urogenital distress inventory. Continence program for women research group. Neurourol Urodyn.

[44] Freeman R, Wieling W, Axelrod FB (2011). Consensus statement on the definition of orthostatic hypotension, neurally mediated syncope and the postural tachycardia syndrome. Clin Auton Res.

[45] Bush K, Kivlahan DR, McDonell MB (1998). The AUDIT alcohol consumption questions (AUDIT-C): an effective brief screening test for problem drinking. Arch Intern Med.

[46] The American Geriatrics Society 2015 Beers Criteria Update Expert Panel (AGS 2015) (2015). American Geriatrics Society 2015 updated beers criteria for potentially inappropriate medication use in older adults. J Am Geriatr Soc.

[47] Böttiger Y, Laine K, Korhonen T, et al. Development and pilot testing of PHARAO – a decision support system for pharmacological risk assessment in the elderly. Eur J Clin Pharmacol. 2017; 10.1007/s00228-017-2391-3. [Epub ahead of print] 10.1007/s00228-017-2391-3 PMC5808089 29198061

[48] Guirquis LM. Pharmacy Patient Care Practice: Focus on Communications in the Theoretical Framework of Pharmaceutical Care. Counseling, Concordance, Communication. Innovative education for pharmacists. 2nd edition. FIP and IPSF 2012. Available at: https://fip.org/files/fip/HaMIS/fip_ipsf_pce_2nd_2012.pdf. Accessed 24 Sept 2017.

